# Dynamic spillovers and portfolio implication between green cryptocurrencies and fossil fuels

**DOI:** 10.1371/journal.pone.0288377

**Published:** 2023-08-03

**Authors:** Zaghum Umar, Sun-Yong Choi, Tamara Teplova, Tatiana Sokolova

**Affiliations:** 1 College of Business, Zayed University, Abu Dhabi, United Arab Emirates; 2 Department of Financial Mathematics, Gachon university, Seongnam, Republic of Korea; 3 Centre for Financial Research & Data Analytics, Faculty of Economic Sciences, HSE University, Moscow, Russia; University of Southampton - Malaysia Campus, MALAYSIA

## Abstract

Are green investments decoupled from the dirty investment such as the fossil fuel markets? We address this issue by extending the literature on environmental, social, and governance (ESG) assets by examining the dynamic relationship between fossil fuels and digital ESG assets proxied by green cryptocurrencies using the TVP-VAR(Time-varying parameter vector auto regression) spillover framework. Furthermore, we analyze the hedging attributes of green cryptocurrencies and fossil fuels in a minimum connectedness framework. The main findings are as follows: First, green cryptocurrencies are the main shock transmitters in all asset systems. Second, the dynamic connectedness between green cryptocurrencies and fossil fuels increased during the COVID-19 and Russia-Ukraine conflicts. Third, green cryptocurrencies have shown considerable hedging effectiveness against the fossil fuels. Our study has important implications for investors, regulators, and policy makers, such as shifting to green cryptocurrencies, regulation of carbon footprint, and promoting eco-friendly assets.

## Introduction

The 2030 Agenda for Sustainable Development was launched by the United Nations Department of Economics and Social Affairs in 2015. In line with this, the need for environmental friendliness is increasing in all industrial fields. Therefore, individual and institutional investors have become interested in an eco-friendly economy and consequently, they have invested heavily in green energy technologies, green assets, and environmentally friendly companies [1]. For example, the global green bond market has grown rapidly since the first issue of green bonds by the European investment bank in 2007. [2, 3]. In particular, the importance of a green economy and green assets in the global financial market has increased due to the COVID-19 pandemic(See “Setting a structural agenda for a green economic recovery from COVID-19”, OECD, Working paper, Nov. 2021).

Similarly, digital assets such as cryptocurrencies have been developing rapidly and have been recognized as important assets by individual and institutional investors. However, many carbon emissions generated in the process of adopting cryptocurrency are causing environmental problems such as global warming and e-waste [4–7]. Accordingly, cryptocurrencies have received considerable criticism for environmental problems, and green(eco-friendly) cryptocurrencies have been developed as an alternative. To circumvent such criticism, green cryptocurrencies use renewable energy, such as solar, hydroelectric, and wind, in the mining process. Therefore, they are called the “green” cryptocurrencies and are more environmentally friendly because they consume less energy than other cryptocurrencies do. Green cryptocurrencies have recently become an important emerging asset for environmentally conscious investors(See “These Green Cryptocurrencies Should Be On Your Radar In 2022”, investing.com).

Climate risk has become a critical consideration for investors and portfolio managers due to its potential impact on investment performance. For example, companies may experience financial losses due to changes in policies and regulations aimed at reducing greenhouse gas emissions. Additionally, investors and portfolio managers have a fiduciary duty to manage risks that could affect the value of their clients’ investments. Climate risk is one such risk that could have a direct impact on the value of investments in companies that rely heavily on fossil fuels, as consumers and governments transition to renewable energy sources. Lastly, considering climate risk in investment decision-making promotes sustainable investing practices that align with ESG considerations.

Motivated by the importance of climate risk in finance, we investigated the relationship between fossil fuels, which are recognized as the main culprit of environmental pollution, and green cryptocurrencies, an eco-friendly asset in the cryptocurrency market. Based on the connectedness between these, we further examined the hedging effectiveness of green cryptos. Furthermore, we constructed a portfolio of green cryptocurrencies and fossil fuels and analyzed their hedging performance. Thus, we employed Brent and WTI(West Texas Intermediate) crude oil, natural gas, and coal prices as indicators of the fossil fuel markets. In addition, five green cryptocurrencies, namely, Cardano, Ripple, IOTA, Stellar, and Nano, were employed [8].

Methodologically, we used the time-varying parameter VAR (TVP-VAR) introduced by [9] based on the spillover framework [10] to examine the relationship between fossil fuels and green cryptos in both static and dynamic ways. In particular, the TVP-VAR method is less sensitive to outliers than the VAR model and avoids the loss of observation during estimation. In addition, we calculate the minimum connectedness portfolio introduced in [11]. Minimum connectedness indicates the level of the portfolio that is less affected by shocks within the portfolio.

However, there are relatively few studies on green cryptocurrency compared with other studies on green assets, such as green bonds and green stock indices. To the best of our knowledge this is the first study to analyze the dynamic spillover and connectedness among green cryptocurrencies and dirty investments such as fossil fuels. Thus, we extend the literature on the decoupling of green investments and dirty investments by taking into account a new emerging digital asset class of green cryptocurrencies. Empirically, we employ a robust methodology that allows us to account for net transmitters and recipient of spillover in these two markets. Furthermore, the computation of minimum connectedness portfolios allows us to provide practical insights for investor and policy makers. Overall, we contribute to the literature by analyzing the linkage between green cryptocurrencies and fossil fuels. In particular, our research is meaningful as a basis for responding to the environmental issues caused by the rapid expansion of the cryptocurrency market.

The remainder of this paper is organized as follows. In the following section, we review the previous studies to our research topic. Section Data and methods explains the data and methodology used. Section Empirical Analysis presents our empirical results. Finally, concluding remarks are presented in the last Section.

## Literature review

The advent of digital assets has led a large strand of literature focused on various aspects of risk and returns of these assets as well as their relationship with other asset classes. [12–16] In the similar vein, an important strand of literature focused on green and sustainable finance is gaining impetus and attracting wide attention. [13, 17–21]. This heightened interest in green investment was also reflected in the digital currency markets with the development of green cryptocurrencies, also known as eco-friendly or sustainable cryptocurrencies. One of the unique features of these cryptocurrencies is their focus on reducing the carbon footprint of cryptocurrency mining and transactions, which have traditionally been associated with high energy consumption and emissions. Given the role of fossil fuels as the predominant source of energy coupled with their adverse impact on the environment, the relationship between green cryptocurrencies and fossil fuel needs an exploration.

The issue of the environmental impact of traditional cryptocurrencies and digital assets has been documented in the literature, with a number of these studies highlighting the phenomenal amount of energy required for cryptocurrency mining activities. [22] documents the massive use of energy in mainstream cryptocurrency mining. [23] document the impact of cryptocurrency on climate change and document that crypto mining induced energy demand has an adverse impact on climate. Similarly, [24] document the adverse impact of the crypto mining activity on environment degradation. [25] developed an index quantifying the effect of environmental attention due to the increasing energy demand and resulting pollution. In contrast, green cryptocurrencies are designed to address these concerns. Their index of environmental uncertainty shows positive effect on various measures of market volatility as well as crude oil. Overall, the existing literature recommends the development of sustainable cryptocurrencies that have lower environmental degradation effects.

Motivated by these studies, a new strand of literature focused on sustainable digital assets emerged. For example, [26] examined long memory, multifractality, and chaoticity in green cryptocurrencies. They document a more profound chaotic behavior of Islamic and green cryptocurrencies. Moreover, there has been an increasing interest in the intersection of green digital assets and the fossil fuel industry. [27] analyzed the dynamic relationships among clean energy, green, and non-green cryptocurrencies. [28] analyzed the connectedness of renewable energy tokens and fossil fuel markets and documents a weak connectedness between these two asset classes. [8] studied the extreme tail dependence of green cryptocurrencies with non-green cryptocurrencies and carbon prices and documents that green crypto currencies are weakly connected with non-green cryptocurrencies. We contribute to this strand of literature by extending the work of [8, 27, 28] by investigating the connectedness dynamics of green cryptocurrencies and fossil fuel markets.

## Data and methods

### Data

**Fig 1:**
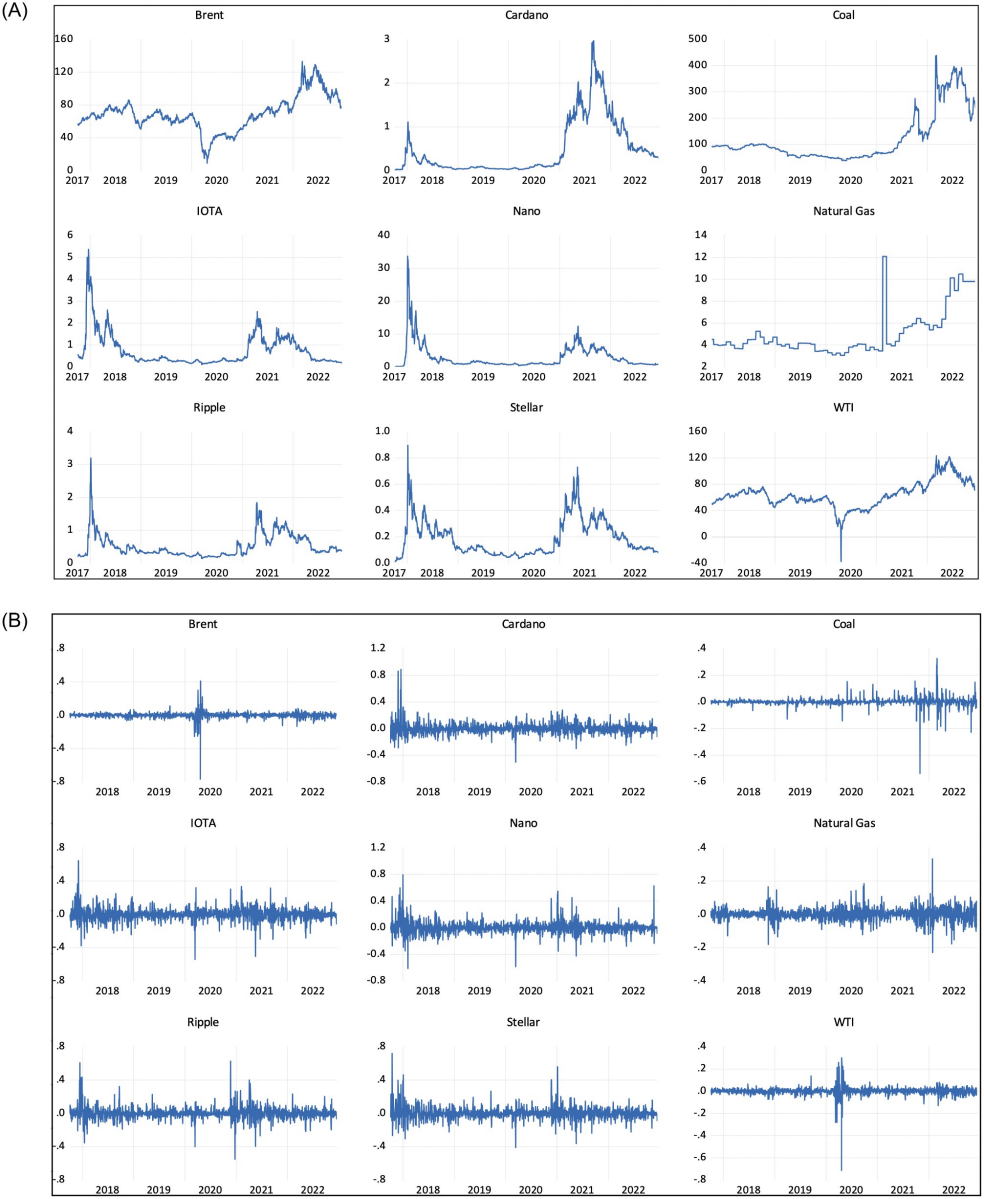
Price and return series. Notes: This figure reports the time series of the assets employed in this study over the sample period. Panel A and Panel B displays the price and returns respectively.

Following [27], we employed five major green cryptocurrencies: Cardano, Ripple, IOTA, Stellar, and Nano. We employed four series of proxies for fossil fuel markets encompassing the crude oil market (both Brent crude and WTI crude), natural gas, and coal. We used the longest available matched time series as the sample period, ranging from October 2017 to December 2022. Data for fossil fuel assets were obtained from Bloomberg, whereas cryptocurrency data were obtained from coinmarketcap.com. The detailed description of all the series is provided in Table 1. This period is characterized by a number of important events affecting both fossil fuel markets as well as cryptocurrencies, such as the boom of cryptocurrencies, the COVID-19 pandemic, and the geopolitical crisis induced by the Russia–Ukraine conflict. We report the sample statistics and pictorial evolution of the series in Table 2 and Fig 1, respectively. We noticed that the average return on the three cryptocurrencies (Cordano, Nano, and Stellar) is higher than the return on fossil fuel markets, whereas IOTA has a negative mean return. The sizably higher risk of cryptocurrencies is evident by the relatively larger standard deviations of cryptocurrencies compared to fossil fuel assets.

**Table 1:** Data description. Notes: This table presents the source, frequency, unit and currency of each variable used in this study.

Variable	Description	Source	Frequency	Unit	Currency
Brent	Brent oil representing fossil fuel market	Bloomberg	daily	Price/return	USD
Cardano	Cardano is a green cryptocurrency	coinmarketcap.com	daily	Price/return	USD
Coal	Coal is a source of fossil fuel	coinmarketcap.com	daily	Price/return	USD
IOTA	IOTA is a green cryptocurrency	coinmarketcap.com	daily	Price/return	USD
Nano	Nano is a green cryptocurrency	coinmarketcap.com	daily	Price/return	USD
Natural Gas	Natural Gas is a fossil fuel source	Bloomberg	daily	Price/return	USD
Ripple	Ripple is a green cryptocurrency	coinmarketcap.com	daily	Price/return	USD
Stellar	Stellar is a green cryptocurrency	coinmarketcap.com	daily	Price/return	USD
WTI	WTI (West Texas Intermediate) oil is a fossil fuel source	Bloomberg	daily	Price/return	USD

**Table 2:** Sample statistics. Notes: This table presents present the sample statistics.

	Brent	Cardano	Coal	IOTA	Nano	Natural Gas	Ripple	Stellar	WTI
Mean	0.0002	0.0017	0.0007	-0.0009	0.0016	0.0006	0.0004	0.0013	0.0003
Median	0.0014	-0.0006	0.0000	-0.0009	0.0000	0.0000	-0.0010	-0.0015	0.0010
Maximum	0.4120	0.8867	0.3262	0.6437	0.7930	0.3328	0.6267	0.7193	0.3002
Minimum	-0.7727	-0.5037	-0.5369	-0.5433	-0.6146	-0.2295	-0.5504	-0.4100	-0.7181
Std. Dev.	0.0387	0.0828	0.0322	0.0771	0.0956	0.0381	0.0738	0.0767	0.0388
Skewness	-5.2175	2.2253	-2.6268	0.1155	1.1540	0.2307	0.9405	1.3947	-4.2390
Kurtosis	136.8320	25.8959	81.1926	11.9418	14.9309	10.3915	17.6071	15.4877	101.6467
Observations	1358	1358	1358	1358	1358	1358	1358	1358	1358

### Methodological framework

We employed the TVP-VAR, extension of the classical [10] framework proposed in [9] because of its desirable attributes, such as no loss of observation from rolling windows and less reliance on outliers. We refer to the [9] for Complete methodological details. The TVP-VAR-based framework boils down to the computation of a connectedness index, given as follows:

$$\begin{array}{c} C_{t}\left(H\right)=\frac{\sum_{i,j=1,i\neq j}^{m}{\tilde{\Phi }}_{ij,t}\left(H\right)}{\sum_{i,j=1}^{m}{\tilde{\Phi }}_{ij,t}\left(H\right)}\times 100=\frac{\sum_{i,j=1,i\neq j}^{m}{\tilde{\Phi }}_{ij,t}\left(H\right)}{m}\times 100. \end{array}$$ (1)

The spillover from asset *i* to all other assets *j*, termed the “TO” directional spillover, is given as

$$\begin{array}{c} C_{i\to j,t}\left(H\right)=\frac{\sum_{j=1,i\neq j}^{m}{\tilde{\Phi }}_{ji,t}\left(H\right)}{\sum_{j=1}^{m}{\tilde{\Phi }}_{ji,t}\left(H\right)}\times 100. \end{array}$$ (2)

The spillover received by asset *i* from all other assets *j*, termed as the “FROM” directional spillover is given as

$$\begin{array}{c} C_{i\leftarrow j,t}\left(H\right)=\frac{\sum_{j=1,i\neq j}^{m}{\tilde{\Phi }}_{ij,t}\left(H\right)}{\sum_{j=1}^{m}{\tilde{\Phi }}_{ij,t}\left(H\right)}\times 100. \end{array}$$ (3)

Finally, to identify an asset as a transmitter of the receiver of spillover, the “NET” directional spillover is given as

$$\begin{array}{c} C_{i,t}\left(H\right)=C_{i\to j,t}\left(H\right)-C_{i\leftarrow j,t}\left(H\right). \end{array}$$ (4)

To compute the portfolio implications and hedging effectiveness of fossil fuels and green cryptocurrencies, we employed a minimum connectedness framework [11] that minimizes spillover due to interconnectedness by incorporating pairwise connectedness such that

$$\begin{array}{c} w_{it}=\frac{PCI_{t}^{-1}I}{IPCI_{t}^{-1}I} \end{array}$$ (5)

where *PCI*^*t*^ denotes the matrix of pairwise connectedness and *I* is the identity matrix.

## Empirical analysis

We start our empirical analysis by discussing the average spillover of fossil fuel and green cryptocurrencies during the sample period, termed static analysis, followed by a time-varying analysis of the spillover.

### Static analysis

We report the average return and volatility connectedness analysis of the fossil fuel markets and green cryptocurrencies during the sample period in Panel A and Panel B of Table 3, respectively. The discussion begins with Panel A, focusing on returns. The overall connectedness of the system is sizable, with a total connectedness index (TCI) value of 49.64%, given in the bottom right edge of Panel A of Table 3. We noticed that cryptocurrencies are the main transmitters of spillovers to the system of all the variables given in the penultimate row of Panel A of Table 3. In particular, Cardano, Ripple, and Stellar exhibit significantly higher spillover transmissions to the system. Among fossil fuel assets, we observed that oil is the major transmitter of spillover, whereas both natural gas and coal exhibit a significantly lower spillover transmission. The spillover received by each variable from the system of all other variables, displayed in the last column of Panel A of Table 3, exhibits a similar pattern to cryptocurrencies receiving sizably higher spillover from the system compare and fossil fuel markets receiving lower levels of spillover. Lastly, to characterize the role of each asset as a net transmitter/receiver of spillovers, we analyzed the last row of Panel A of Table 3. We found that all cryptocurrencies (with the exception of Nano) are net transmitters of spillovers, whereas all fossil fuel assets are recipients of spillovers. Overall, Cardano is the highest transmitter of spillover (highest value), whereas natural gas is the highest recipient of spillover (lowest value).

**Table 3:** Static spillover. Notes: This table reports the static spillovers of assets. TCI denotes the total connectedness index.

Panel A: Returns										
	WTI	Brent	Natural Gas	Coal	Cardano	Ripple	IOTA	Stellar	Nano	FROM
WTI	55.34	32.21	1.57	2.12	1.76	1.51	1.88	1.87	1.74	44.66
Brent	33.12	55.18	1.46	2.16	1.87	1.33	1.56	1.69	1.62	44.82
Natural Gas	2.66	2.19	84.13	2.94	1.91	1.59	1.25	1.64	1.7	15.87
Coal	2.83	2.69	2.91	85.61	1.54	0.95	1.35	1.03	1.08	14.39
Cardano	0.66	0.66	0.75	0.62	33.1	16.65	16.51	18.03	13.03	66.9
Ripple	0.67	0.61	0.59	0.41	16.96	33.81	15.11	18.73	13.11	66.19
IOTA	0.91	0.73	0.56	0.58	17.16	15.55	35.06	15.82	13.61	64.94
Stellar	0.77	0.69	0.61	0.4	18.17	18.47	15.02	32.49	13.37	67.51
Nano	0.92	0.72	0.7	0.57	14.74	14.34	14.75	14.73	38.53	61.47
TO	42.54	40.48	9.16	9.81	74.12	70.39	67.45	73.54	59.27	TCI
NET	-2.13	-4.33	-6.71	-4.58	7.22	4.2	2.51	6.03	-2.2	49.64
Panel B: Volatility										
	WTI	Brent	Natural Gas	Coal	Cardano	Ripple	IOTA	Stellar	Nano	FROM
WTI	57.25	26.49	3.05	4.41	1.92	1.79	1.77	1.81	1.52	42.75
Brent	27.1	55.73	2.63	4.87	2.14	1.95	1.83	1.89	1.87	44.27
Natural Gas	4.6	3.81	73.54	7.02	2.27	2.16	2.33	2.26	2.01	26.46
Coal	3.79	3.9	5.3	75.56	3.18	2.08	1.96	1.91	2.33	24.44
Cardano	1.34	1.48	1.18	2.32	37.45	13.78	14.59	16.26	11.61	62.55
Ripple	1.52	1.52	1.48	2.32	14.19	39.06	12.55	16.35	11.01	60.94
IOTA	1.62	1.57	1.16	1.8	15.11	12.49	41.01	13.18	12.06	58.99
Stellar	1.39	1.58	1.19	1.87	16.29	16.15	12.79	36.35	12.38	63.65
Nano	1.54	1.28	1.43	2.99	12.9	11.27	12.97	13.48	42.13	57.87
TO	42.9	41.62	17.42	27.6	68	61.67	60.79	67.13	54.79	TCI
NET	0.15	-2.65	-9.04	3.16	5.45	0.73	1.8	3.48	-3.08	49.1

The volatility connectedness estimated in Panel B of Table 3 differs from the patterns exhibited by the return connectedness estimates. While most cryptocurrencies still hold their role as major transmitters of spillover, we noticed that the volatility of coal seems to be an influential net transmitter of spillover. This is particularly important, given that coal is widely considered the most environmentally harmful fossil fuel.

**Fig 2:**
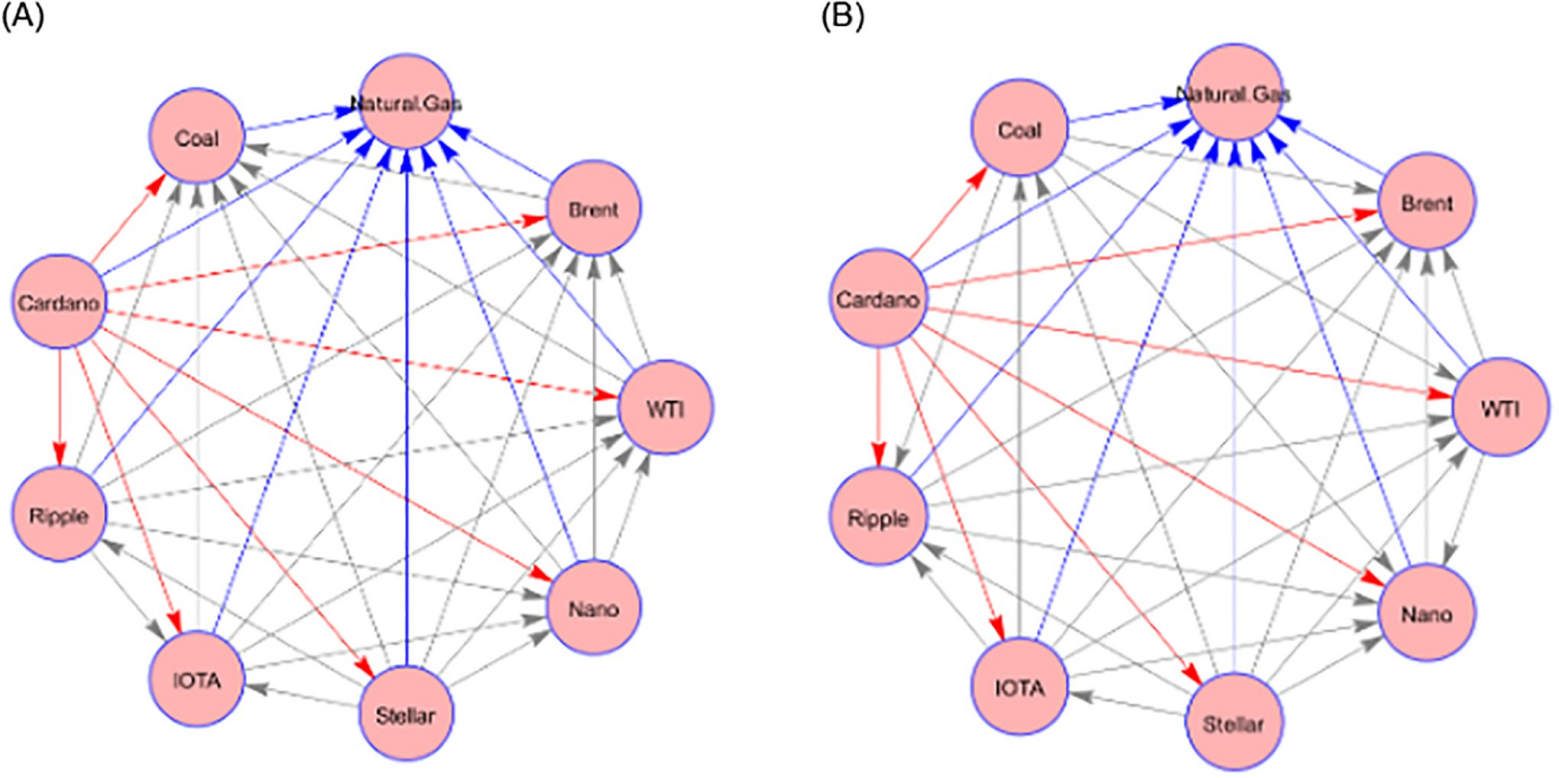
The pairwise spillover in the form of the network graph. Notes: This figure reports the pairwise connectedness of the assets employed.

The off-diagonal entries in Table 3 show the pairwise spillovers of each pair of assets in the system. However, for ease of analysis, we report pairwise spillover in the form of the network graph shown in Fig 2. The direction of the arrow represents the transmission of pairwise spillovers from the source to the edge. Additionally, we can assess the centrality of a particular variable in the system using the number of arrows stemming from that variable, and vice versa. The pairwise return spillovers of both return and volatility show the central role of Cardano as the main transmitter of pairwise spillover to all other assets, and natural gas as the recipient of spillover from all other assets.

### Dynamic analysis

**Fig 3:**
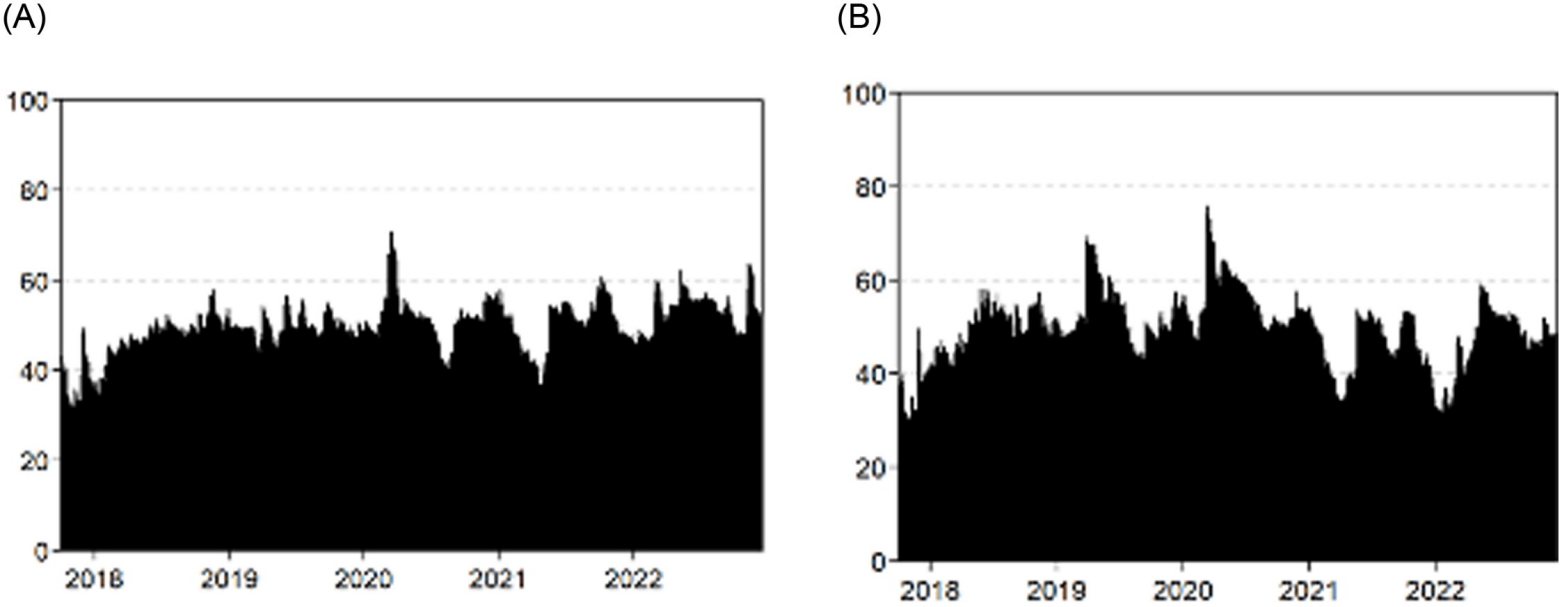
The return and volatility total connectedness. Notes: This figure reports the total connectedness index (TCI) of the assets employed over the sample period.

We report a time-varying dynamic analysis of the spillovers over the sample period. We start with the return and volatility total connectedness analyses depicted in Panels A and B of Fig 3, respectively. The total connectedness varies over the sample period, with values ranging between 30% and 75%, thus underscoring the importance of time-varying spillover analysis. Connectedness tends to increase during the period of crisis, with the highest value at the peak of the COVID-19 pandemic, followed by the Russia–Ukraine conflict in February 2022.

**Fig 4:**
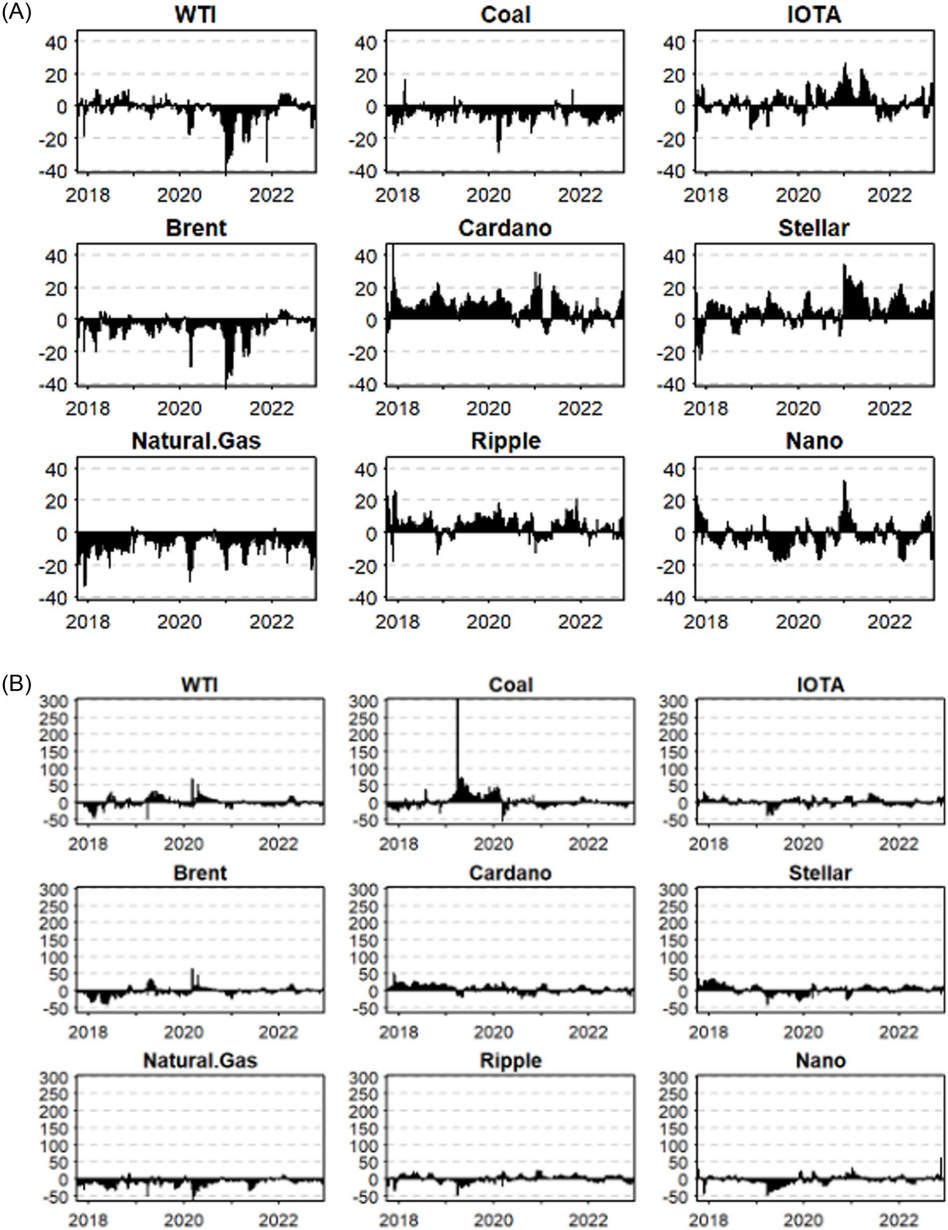
The time-varying net connectedness. Notes: This figure reports the net connectedness analysis of the assets employed over the sample period.

To gain in-depth insight into the dynamics of the role of each asset as a transmitter and recipient of spillovers during the sample period, we report the time-varying net connectedness of each asset’s returns and volatility in panels A and B of Fig 4. We start our discussion with the return analysis in Panel A. Among the crypto assets, we found that Cardano is predominantly a net transmitter of spillovers, except for very short intervals. Among the other crypto-currencies, Ripple, Stellar, and IOTA exhibit alternating patterns, with more frequent switching for IOTA. Lastly, Nano seems to be predominantly a recipient of spillovers across most of the sample period. The diverse behavior of cryptocurrencies across the sample period underscores the importance of analyzing them as unique assets. Among fossil fuel assets, coal and natural gas are predominantly net recipients of return spillover throughout the sample period, where WTI exhibits some alternating patterns. Interestingly, both the WTI and Brent exhibited a net transmission of spillover in the wake of the Russia–Ukraine conflict, underscoring the importance of oil as an important energy source. Regarding the net volatility spillover exhibited in Panel B, we noticed certain unique patterns that were not reflected in the return analysis. The most noticeable pattern was that all cryptocurrencies, except Stellar, are recipients of volatility spillovers in the wake of the Russia–Ukraine conflict. Another pattern compared to return connectedness was that fossil fuel assets, namely crude oil and coal, exhibit intervals of net transmission of spillover during the sample period. Coal has an extended period of volatility transmission, which ended with the advent of the COVID-19 pandemic. On the contrary, we noticed that oil is a major transmitter of volatility spillover for the periods of the COVID-19 as well as the Russia–Ukraine conflict.

### Portfolio and hedging implications

Finally, we discuss the portfolio implications of the connectedness analysis. We consider an investor whose asset menu includes both fossil fuel and green cryptocurrencies. Table 4 reports the average minimum connectedness portfolio weights, their respective standard deviations, and the hedging effectiveness of each of the fossil fuel and green crypto assets. As expected, natural gas and coal have the highest weight because of their relative disentanglement from other assets. The highest hedging effectiveness is observed for cryptocurrencies. Overall, an investor investing in both fossil fuels and green cryptocurrencies provides substantial hedging effectiveness in the portfolio context.

**Table 4:** Portfolio analysis. Notes: This table reports the average and standard deviation (SD), minimum connectedness portfolio weight of each asset, and hedging effectiveness (HE). * denotes 1% level of significance.

	Mean	Std.Dev.	HE
WTI	0.12	0.03	0.60*
Brent	0.12	0.03	0.60*
Natural Gas	0.2	0.02	0.52*
Coal	0.2	0.02	0.44*
Cardano	0.03	0.04	0.81*
Ripple	0.06	0.05	0.83*
IOTA	0.08	0.04	0.83*
Stellar	0.08	0.05	0.81*
Nano	0.11	0.04	0.87*

## Discussion and conclusion

In this study, we investigated the dynamic relationship between fossil fuels and green cryptocurrencies using the TVP-VAR spillover framework. Furthermore, based on this spillover approach, we analyzed the hedging performance. Our main findings are as follows:

First, we notice that there is sizable connectedness between green cryptocurrencies and fossil fuels, thus implying that on a system wide basis these two markets are not decoupled. These results are to the findings of [8, 22, 25, 29] who documented higher system connectedness between socially responsible investments and fossil fuel markets for different asset classes. However, contrary to above studies we document that green cryptocurrencies are the main shock transmitters in all asset systems. In particular, Cardano and natural gas are the major transmitters and recipients of pairwise spillovers, respectively. These results were confirmed by both the static and dynamic analyses. The differences in the behavior of various cryptocurrencies are also documented by [30].

Second, the dynamic connectedness between green cryptocurrencies and fossil fuels increased during the crisis, with the highest value occurring during the COVID-19 and Russia–Ukraine (RU) conflict. These results are also shown in other studies, underscoring the close monitoring of portfolios during such periods of turmoil [31–33]. Furthermore, consistent with existing literature, we note that the volatility connectedness exhibits a higher increase relative to return connectedness during periods of turmoil. [34]

Third, during the RU conflict, most cryptocurrencies were recipients of volatility spillovers, while oil was a major volatility transmitter. One of the reasons for this result is that the RU conflict negatively impacted cryptocurrency investors seeking liquidity, causing a sharp drop in the green and non-green cryptocurrency market [35]. In addition, oil had a significant impact on other assets and disrupted the supply of crude oil from Russia; oil became a volatility transmitter during the war [36]. Finally, based on the spillover results of fossil fuels and green cryptocurrencies, they show considerable hedging effectiveness.

While we have analyzed the connectedness and portfolio dynamics using a novel time-based approach, we did not explicitly account for various investment horizons for investors as pointed out by [37–39]. However, we leave this issue as a future extension of our study. Towards this end, time-frequency domain methods may be used. Another potential extension of our work is to use a broader array of green, Islamic and conventional cryptocurrencies and analyze their spillover dynamics with fossil fuels as well as renewable fuels.

Climate change is a global problem that affects various industries, and it is important for investors and portfolio managers to consider the potential risks and opportunities associated with it when making investment decisions. Therefore, the results of our study have important implications for investors, policymakers, and portfolio managers. By using this information, investors can make more informed decisions, possibly reducing their exposure to fossil fuels and shifting towards environmentally friendly assets, like green cryptocurrencies. Furthermore, policymakers can also encourage the development and adoption of eco-friendly assets, such as green cryptocurrencies, as they could help to reduce the overall carbon footprint of the financial industry. Additionally, policymakers could create regulations to address the significant carbon footprint issues caused by the cryptocurrency market. This study also serves as a wake-up call for businesses to recognize the importance of environmental responsibility and sustainability and take steps to reduce their carbon footprint.
